# Knockout of the Mitochondrial Calcium Uniporter Strongly Suppresses Stimulus-Metabolism Coupling in Pancreatic Acinar Cells but Does not Reduce Severity of Experimental Acute Pancreatitis

**DOI:** 10.3390/cells9061407

**Published:** 2020-06-05

**Authors:** Michael Chvanov, Svetlana Voronina, Xiaoying Zhang, Svetlana Telnova, Robert Chard, Yulin Ouyang, Jane Armstrong, Helen Tanton, Muhammad Awais, Diane Latawiec, Robert Sutton, David N. Criddle, Alexei V. Tepikin

**Affiliations:** 1 Department of Cellular and Molecular Physiology, University of Liverpool, Liverpool L69 3BX, UK; s.g.voronina@liverpool.ac.uk (S.V.); stelnova@liverpool.ac.uk (S.T.); rpchard@liverpool.ac.uk (R.C.); yulinouyang@gmail.com (Y.O.); HelenE_Tanton@DFCI.HARVARD.EDU (H.T.); criddle@liverpool.ac.uk (D.N.C); 2 Liverpool Pancreatitis Research Group, Royal Liverpool University Hospital, Institute of Translational Medicine, University of Liverpool, Liverpool L69 3BX, UK; stefzxy@hotmail.com (X.Z.); janearm@liverpool.ac.uk (J.A.); awais@liverpool.ac.uk (M.A.); latawiec@liverpool.ac.uk (D.L.); sutton@liverpool.ac.uk (R.S.)

**Keywords:** acute pancreatitis, Ca^2+^ signaling, mitochondrial calcium uniporter, pancreatic acinar cells

## Abstract

Acute pancreatitis is a frequent disease that lacks specific drug treatment. Unravelling the molecular mechanisms of acute pancreatitis is essential for the development of new therapeutics. Several inducers of acute pancreatitis trigger sustained Ca^2+^ increases in the cytosol and mitochondria of pancreatic acinar cells. The mitochondrial calcium uniporter (MCU) mediates mitochondrial Ca^2+^ uptake that regulates bioenergetics and plays an important role in cell survival, damage and death. Aberrant Ca^2+^ signaling and mitochondrial damage in pancreatic acinar cells have been implicated in the initiation of acute pancreatitis. The primary aim of this study was to assess the involvement of the MCU in experimental acute pancreatitis. We found that pancreatic acinar cells from MCU^−/−^ mice display dramatically reduced mitochondrial Ca^2+^ uptake. This is consistent with the drastic changes of stimulus-metabolism coupling, manifested by the reduction of mitochondrial NADH/FAD^+^ responses to cholecystokinin and in the decrease of cholecystokinin-stimulated oxygen consumption. However, in three experimental models of acute pancreatitis (induced by caerulein, taurolithocholic acid 3-sulfate or palmitoleic acid plus ethanol), MCU knockout failed to reduce the biochemical and histological changes characterizing the severity of local and systemic damage. A possible explanation of this surprising finding is the redundancy of damaging mechanisms activated by the inducers of acute pancreatitis.

## 1. Introduction

Acute pancreatitis (AP) is a frequent disease of considerable morbidity [1,2]. Mitochondrial dysfunction has been shown to play an important role in death/damage of pancreatic acinar cell and the initiation of AP [3,4,5,6]. In this study, we focused on the contribution of mitochondrial Ca^2+^ entry to the severity of this pancreatic disease. The important secretagogues acetylcholine and cholecystokinin (CCK) utilize Ca^2+^ signaling cascade to trigger and regulate secretion of digestive enzymes and precursors of digestive enzymes from pancreatic acinar cells (reviewed in [7,8,9]). Cytosolic Ca^2+^ signals are produced as a result of Ca^2+^ release from the intracellular stores (primarily the endoplasmic reticulum (ER)) and Ca^2+^ entry via the store-operated Ca^2+^ channels (SOCS) in the plasma membrane (PM) [7,8,9]. Cytosolic Ca^2+^ responses in the acinar cells are accompanied by Ca^2+^ entry into mitochondria and changes in mitochondrial Ca^2+^ concentration ([Ca^2+^]m) [10,11]. Similar to other cell types (e.g., [12]), Ca^2+^ entry into mitochondria mediates stimulus–metabolism coupling in the acinar cells [11,13]. While physiological Ca^2+^ oscillations are vital for the normal functioning of pancreatic acinar cells (reviewed in [7,8,9]), aberrant sustained Ca^2+^ responses are considered as important early events in the development of the cell damage and initiation of acute pancreatitis [14,15,16,17,18,19,20]. Excessive increase of cytosolic Ca^2+^ was associated with several damaging downstream responses in the acinar cells pertinent to the induction of acute pancreatitis. These downstream responses include activation of calpains [21], vacuolization [22,23,24,25], activation of calcineurin [26,27], intracellular trypsinogen activation [28,29,30] and aberrant autophagy [31,32]. It has been extensively documented that mitochondrial overload with calcium can precipitate apoptotic and/or necrotic cell death [33,34,35,36]. In particular, mitochondrial calcium overload was suggested as an important mediator of Ca^2+^ toxicity in the acinar cells [6,37,38,39]. Mitochondrial calcium uniporter (MCU) is the principal channel-forming component of the machinery for calcium entry into mitochondria ([40,41], recently reviewed in [42]). Preventing mitochondrial Ca^2+^ entry via MCU was shown to protect a number of tissues and cell types against injurious stimuli: genetic ablation of MCU has been shown to protect neurons against excitotoxicity-induced injury and death [43,44,45,46], reduce apoptosis of airway epithelial cells [47], protect cardiac tissue against hypoxia–reperfusion injury [48] and reduce the pro-inflammatory responses in various models of inflammation [49,50,51,52]. We therefore hypothesized that eliminating the main mitochondrial Ca^2+^ entry pathway would reduce the severity of experimental AP and tested this hypothesis using three established AP models in MCU knockout mice.

## 2. Materials and Methods

### 2.1. Animals and Cells

All animal studies were ethically reviewed and conducted according to UK Animals (Scientific Procedures) Act 1986, approved by UK Home Office (PPL 70/8109, renewed as PDC14C46E) and the University of Liverpool Animal Welfare Committee and Ethical Review Body (AWERB). The breeding pairs of heterozygous (MCU^+/−^) mice were generously provided by Dr. Finkel (Centre for Molecular Medicine, NHLBI, National Institute of Health, Bethesda, MD, US). MCU-deficient mice (MCU^−/−^) [53] were generated by crossing MCU^+/−^ mice. The genotype of the mice was confirmed as described in [53]. Wild type (MCU^+/+^) mice utilized in our experiments were littermates of MCU^−/−^ mice.

Animals were maintained at 22 ± 2 °C and exposed to a 12 h light–dark cycle. Prior to experiments, mice had ad libitum access to food and water. Pancreatic acinar cells were isolated by digestion with purified collagenase (200 U/mL) as previously described [30] and kept in extracellular solution containing 140 mM NaCl, 4.7 mM KCl, 1.13 mM MgCl_2_, 1.2 mM CaCl_2_, 10 mM D-glucose and 10 mM 4-(2-hydroxyethyl)-1-piperazineethanesulfonic acid (HEPES) at pH 7.4.

### 2.2. Materials

Propidium iodide (PI), CellEvent™ Caspase-3/7, fura-2-AM and SYTOX^TM^ Red were from Thermo Fisher Scientific (Waltham, MA, US). Boc-Gln-Ala-Arg-MCA was from Enzo Life Sciences (New York, NY, USA). Protease inhibitors were from Roche GmbH (Mannheim, Germany). MEM NEAA solution (100×) was from Gibco/Thermo Fisher Scientific. If not otherwise indicated, other reagents were from Sigma-Aldrich (Dorset, UK). Mitochondria-targeted fluorescent Ca^2+^ probe MtRCaMP was described in [54]. Replication deficient adenovirus for the expression of MtRCaMP in pancreatic acinar cells was a gift from Prof. György Hajnóczky; the virus was amplified by Vector Biolabs (Malvern, Pennsylvania, PA, USA).

### 2.3. Ca^2+^ Measurements

For measurements of cytosolic Ca^2+^ concentration ([Ca^2+^]_C_), pancreatic acinar cells were loaded in darkness with fura-2-AM (5 μM) for 60 min at room temperature and washed in the indicator-free extracellular solution 30 min prior to the beginning of an experiment. Fluorescence images were captured using the Till Photonics Imaging System, based on an Olympus IX71 inverted microscope using 10× objective (NA = 0.4, dry). Excitation wavelengths of 340 and 380 nm were used; emission was recorded with 510 nm filter. Background subtraction was carried out independently at each of the two excitation wavelengths and the ratios of fluorescence (F 340/380) were calculated for each image. 

For adenoviral transfection, required for MtRCaMP expression, cells were seeded on 35-mm glass-bottom dishes (MatTek Corporation, Ashland, MA) pre-coated with poly-l-lysine and infected with the replication-deficient adenovirus at concentration of 6.5 × 10^7^ PFU/mL for 16–18 h at 35 °C in extracellular solution supplemented with 1 mM Na_3_PO_4_, 2 mM sodium pyruvate, 0.02% soybean trypsin inhibitor, MEM NEAA 1% (*v/v*), 100 U/mL penicillin and 100 μg/mL streptomycin. For [Ca^2+^]m measurements, the confocal images of MtRCaMP-transfected cells were taken using a Leica SP2 AOBS system with a 63× objective (NA = 1.4, oil). Excitation wavelength was 594 nm, and the emission bandwidth was 620–700 nm.

### 2.4. NADH and FAD^+^ Fluorescence Measurements

NADH and FAD^+^ autofluorescence of pancreatic acinar cells was measured using a Zeiss 710 confocal microscope equipped with a 40× objective (NA = 1.3, oil). Fluorescence of NADH was excited with a 355 nm laser line and its emission was collected in 400–500-nm bandwidth. Fluorescence of FAD^+^ was excited using 458 nm and its emission was collected in 510–600-nm bandwidth. Sequential excitation for NADH and FAD^+^ was used in these experiments. Images for each fluorophore were collected every 10 s. Background signals (recoded from cell-free areas) were subtracted from the fluorescence signals. Cellular fluorescence signals for all time points were normalized by the values of fluorescence recorded just before CCK addition.

### 2.5. Oxygen Consumption Measurements

The cellular bioenergetics (oxygen consumption rate, OCR) of the isolated cells were determined using a Seahorse XF24 analyzer (Agilent, Boston, MA, USA) as reported previously [55]. The optimum number of cells/well for detection of changes in OCR was determined to be 75,000. Prior to bioenergetics measurements, the isolation medium was changed to Dulbecco’s modified Eagle’s medium (DMEM, pH 7.4) supplemented with 10 mM glucose, 2 mM L-glutamine and 2 mM sodium pyruvate (Sigma-Aldrich, Gillingham, UK). The basal OCR in pancreatic acinar cells was first measured in the absence of any additional stimulation to obtain steady-state, resting levels of respiration. A mitochondrial respiratory function was probed using Agilent Seahorse XF Cell Mito Stress Test (referred to as Mito Stress Test in the other parts of this paper). The Mito Stress Test experiment involved sequential additions of oligomycin, trifluoromethoxy carbonylcyanide phenylhydrazone (FCCP), antimycin A and rotenone; these compounds were injected sequentially through ports of the Seahorse Flux Pak cartridges to achieve the final concentrations of 1, 0.3, 1 and 1 µM, respectively (see https://www.agilent.com/cs/library/usermanuals/public/XF Cell Mito Stress Test Kit User Guide.pdf for further information about the Mito Stress Test). The OCR results of each experiment were normalized to the protein content of the wells determined by BCA assay. 

The OCR measurements were conducted on unstimulated pancreatic acinar cells and the cells stimulated by CCK (CCK was added after the 5th OCR reading, prior to the Stress Test).

### 2.6. Cell Death Assays

Fluorescence of the probes characterizing apoptosis or necrosis was measured on a POLARstar Omega Plate Reader (BMG Labtech, Germany) at 37 °C using 96-well flat bottom plates as previously described [56]. Necrosis of the cells was measured using the fluorescent dye propidium iodide (PI, 10 µg/mL final concentration) or SytoxRed (5 nM, used instead of PI only in the experiments involving TLCS because of observed PI interaction with TLCS in the extracellular solution). Excitation/emission filters of 540/620 nm were used in these experiments. Notably, PI and SytoxRed determine total cell death involving permeabilization of the plasma membrane; this can include primary necrosis, secondary necrosis and regulated necrosis (e.g., necroptosis) (reviewed in [57,58,59]). The relationships between these mechanisms in acinar cells treated with inducers of acute pancreatitis are complex and incompletely understood. Defining such mechanisms was not a primary objective of our study. In our manuscript, we use the term “necrosis” to refer to all possible necrotic processes resulting in the plasma membrane permeabilization (i.e., processes which are resolved by changes in the PI or SytoxRed fluorescence). 

Apoptosis of the acinar cells was measured using CellEvent™ Caspase-3/7 at 1:100 dilution. Fluorescence developed as the result of caspase activation was measured using excitation 480 nm and emission 520 nm. Apoptosis and necrosis measurements were run in triplicates. The end-point readings of necrosis and apoptosis were done at 13 h. All fluorescence measurements for necrosis and apoptosis of the cells from MCU^−/−^ mice were normalized to the corresponding measurements conducted on the cells from MCU^+/+^ mice in the same experiments.

### 2.7. Experimental AP Models

AP was induced by: (1) seven intraperitoneal injections of caerulein (50 μg/kg) at hourly intervals (CER-AP) (see [60] for further information about this AP model); (2) retrograde perfusion of the pancreatic duct with 3 mM TLCS (TLCS-AP) as described in [15,56]; and (3) two hourly intraperitoneal injections of 1.35 g/kg ethanol and 150 mg/kg palmitoleic acid (POA) as described in [6], and this type of experimental pancreatitis is abbreviated as FAEE-AP. The numbers of animals utilized in AP models are described in the Results Section. In control experiments (conducted for each AP model), mice received saline injection/perfusion under the same condition but without an AP inducer (≥4 mice were used in each control experiment). For the CER-AP and its controls, mice were humanly sacrificed at 12 h after the first caerulein injection; for TLCS and FAEE-AP and the relevant controls, mice were humanly sacrificed at 24 h post-induction.

### 2.8. Histopathology 

Pancreatic tissue was fixed in 10% formalin, paraffin-embedded and H&E stained. Pancreatic histopathological scoring was performed on 10 random fields (magnification 200×) by two independent investigators who were unaware of the study design, grading 0–4 was utilized for edema, inflammatory cell infiltration and acinar necrosis, respectively [61]. The overall pancreatic histopathology score was the sum of the individual scores.

### 2.9. Immunoblotting

Pancreatic samples were homogenized on ice in RIPA buffer. The protein concentration of lysed samples was determined using Pierce BCA protein assay kit (Thermo Fisher Scientific). BisTris NuPAGE gels (4–12%; Invitrogen) were used for electrophoresis (30 μg of protein/lane) with MES SDS NuPAGE running buffer (Invitrogen). SeeBlue Plus2 molecular weight standards (Invitrogen) were employed. Proteins were transferred to Hybond-ECL nitrocellulose membrane (Amersham Biosciences). Blots were blocked for 1 h at room temperature in 5% milk dissolved in PBS with 0.05% Tween-20, pH 7.4. Primary antibodies used: HPA016480 (anti-MCU, rabbit poly-clonal, Sigma) 1:400 dilution; C4731 (anti-calnexin, rabbit poly-clonal, Sigma) 1:2000; and A6154 (HRP-conjugated goat anti-rabbit polyclonal, Sigma) 1:1000. The chemiluminescence signal was detected using Pierce Supersignal West Pico chemiluminescent reagents (Thermo Fisher Scientific) with a ChemiDoc XRS + molecular imaging system (Bio-Rad, Hercules, CA, USA).

### 2.10. Enzyme Activity and IL-6 Measurement

Pancreatic trypsin activity was measured using Boc-Gln-Ala-Arg-MCA substrate (380/440 nm excitation/emission) as described previously [62]. Myeloperoxidase activity was determined using tetramethylbenzidine substrate as previously described [63]. Serum amylase and IL-6 were determined kinetically using a Roche automated clinical chemistry analyzer (GMI, Leeds, UK) and ELISA kit (R&D Systems, Abingdon, UK), respectively.

### 2.11. Statistical Analysis

The data were tested for normality using a Shapiro–Wilk test. If a normality hypothesis could not be rejected, further comparisons were made using a two-tailed Student’s t-test. Mann–Whitney test was used to compare two groups of independent observations for the data not following a normal distribution. Statistical significance was set at *p* < 0.05 and indicated by asterisk (*) on the figures. 

## 3. Results

### 3.1. MCU Knockout Suppresses Mitochondrial Ca^2+^ Responses and Its Downstream Effects 

**Figure 1:**
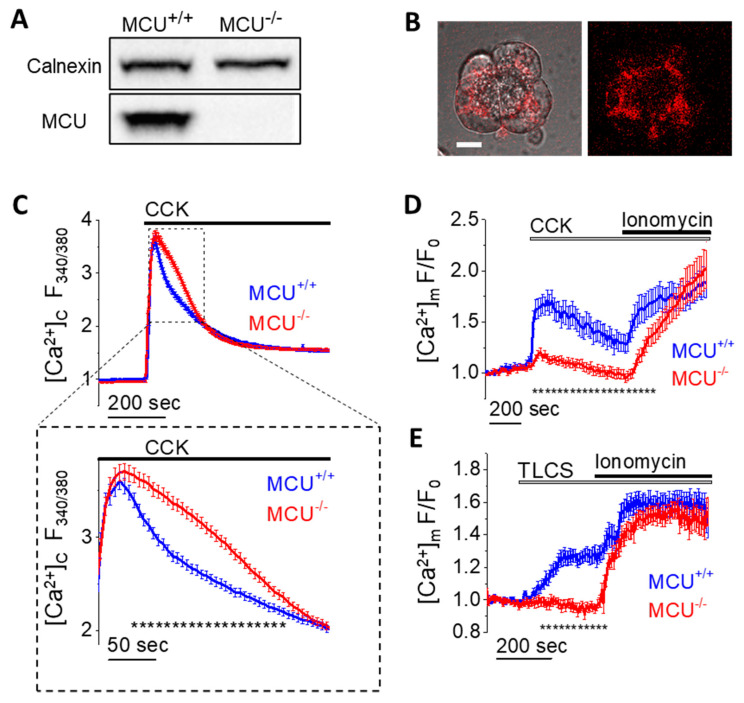
Cytosolic and mitochondrial Ca^2+^ signaling in pancreatic acinar cells from MCU^−/−^ and WT (MCU^+/+^) mice. (**A**) Western blot analysis of MCU in pancreata isolated from MCU^−/−^ and MCU^+/+^ mice. The complete Western blot associated with this figure is shown in Figure S1A. (**B**) Images of MtRCaMP in a small cluster of WT pancreatic acinar cells showing a typical mitochondrial distribution (e.g., [11]). The right panel shows the distribution of fluorescence. The left panel shows the overlay of transmitted light and fluorescence. Scale bar represents 10 µm. A similar distribution was observed in the acinar cells from MCU^−/−^ mice (Figure S1B). (**C**) [Ca^2+^]_C_ responses (measured with fura-2 (loaded in fura-2 AM form), 340 nm:380 nm ratio) to 1 nM CCK in pancreatic acinar cells isolated from MCU^−/−^ mice (*n* = 286 cells, N = 4 mice) and MCU^+/+^ mice (*n* = 197 cells, N = 4 mice). The expanded fragment (lower panel in (**C**)) highlights the period of [Ca^2+^]_C_ responses in which there were significant differences between measurements conducted on acinar cells isolated from MCU^−/−^ mice and MCU^+/+^ mice. Dotted line under the traces (composed of small asterisks) indicates the only period (from 55 to 215 s following CCK addition) when the measurements from MCU^+/+^ and MCU^−/−^ cells were significantly different (*p* < 0.05). Here and in (**D**,**E**) data are presented as the mean value ± standard error of the mean. (**D**) [Ca^2+^]m responses to 1nM CCK followed by 20 µM Ionomycin/10 mM Ca^2+^ in pancreatic acinar cells isolated from MCU^−/−^ mice (*n* = 53 cells, N = 5 mice) and MCU^+/+^ mice (*n* = 25 cells, N = 3 mice). Here and in (**E**) the traces show the fluorescence of MtRCaMP (F) normalized to its initial fluorescence (F_0_). Dotted line under the traces (composed of small asterisks) indicates the period (from 15 to 795 s following CCK addition) when the measurements from MCU^+/+^ and MCU^−/−^ cells were significantly different (*p* < 0.05). (**E**) [Ca^2+^]m responses to 500 µM TLCS followed by 20 µM Ionomycin/10 mM CaCl_2_ in pancreatic acinar cells isolated from MCU^−/−^ mice (*n* = 25 cells, N = 3 mice) and MCU^+/+^ mice (*n* = 22 cells, N = 3 mice). Dotted line under the traces (composed of small asterisks) indicates the period (from 77 to 320 s following TLCS addition) when the measurements from MCU^+/+^ and MCU^−/−^ cells were significantly different (*p* < 0.05).

First, we investigated the effects of MCU knockout on cytosolic and mitochondrial Ca^2+^ responses to known AP inducers. The knockout of MCU was confirmed by Western Blot analysis (Figure 1A and Figure S1A). Cytosolic Ca^2+^ responses ([Ca^2+^]_C_) were measured using a common ratiometric Ca^2+^ indicator fura-2 [64]. To monitor mitochondrial Ca^2+^ responses ([Ca^2+^]m), pancreatic acinar cells from MCU^−/−^ and wild type (WT) mice were transfected with a genetically-encoded fluorescent mitochondrial calcium sensor MtRCaMP [54] (Figure 1B and Figure S1B). The [Ca^2+^]_C_ responses to 1 nM CCK-8 had similar peak amplitudes in pancreatic acinar cells from MCU^−/−^ and WT mice (Figure 1C). The plateau levels at the end of the recording periods were also similar (Figure 1C). There was a small but resolvable difference in the [Ca^2+^]_C_ responses for the time period from 55 to 215 s (Figure 1C) following CCK application. During this period cytosolic Ca^2+^ levels were higher in cells from MCU^−/−^ animals (Figure 1C). 

In contrast to [Ca^2+^]_C_ responses, mitochondrial Ca^2+^ responses to CCK were dramatically reduced in the cells from MCU^−/−^ mice, leaving a very small though resolvable [Ca^2+^]m transient (Figure 1D). 

Application of a high concentration (20 µM) of ionomycin, which increases permeability of the plasma membrane and inner mitochondrial membrane to Ca^2+^, in the presence of 10 mM extracellular Ca^2+^, triggered a rise in [Ca^2+^]m in cells from MCU^−/−^ and MCU^+/+^ mice, confirming the presence and functionality of the mitochondrial Ca^2+^ sensor in pancreatic acinar cells isolated from both strains of mice (Figure 1D). 

Stimulation of the acinar cells with bile acid TLCS 500 µM (Figure 1E) also produced a much greater increase in [Ca^2+^]m in cells from MCU^+/+^ than MCU^−/−^ mice. Stimulation with 200 µM of free palmitoleic acid (POA) produced only a very minor rise of MtRCaMP fluorescence in both MCU^−/−^ and MCU^+/+^ cells (Figure S2). 

**Figure 2:**
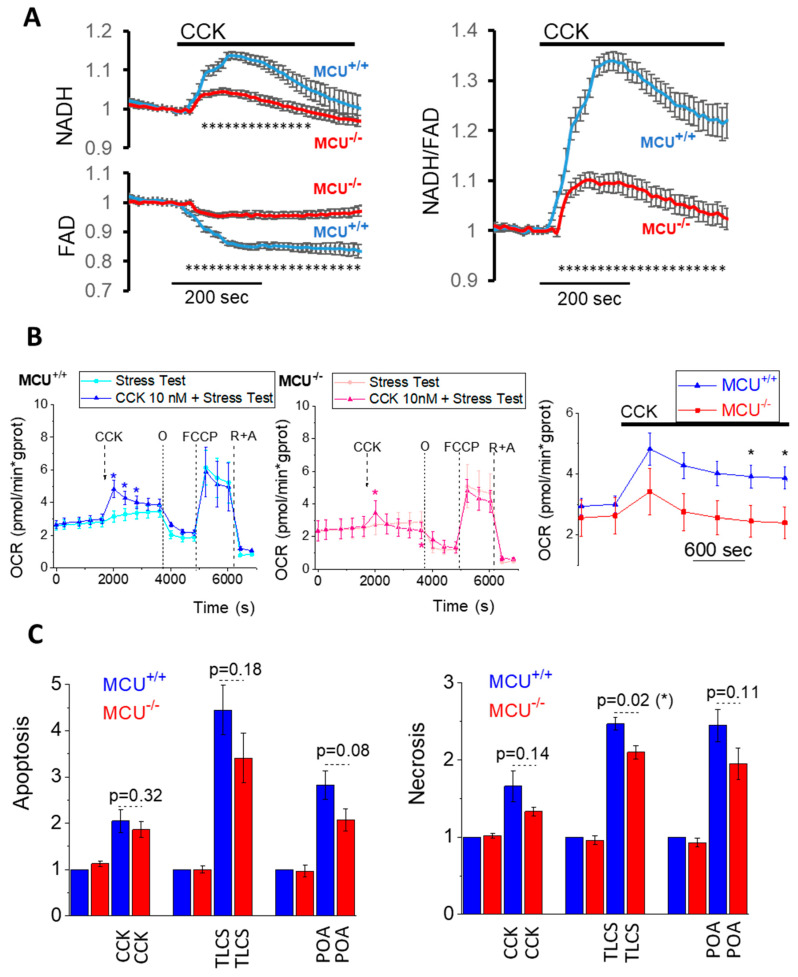
Effect of MCU knockout on NADH/FAD responses, oxygen consumption and cell death of isolated pancreatic acinar cells. (**A**) The left panels show NADH (top) and FAD^+^ (bottom) responses to 1 nM CCK in pancreatic acinar cells isolated from MCU^−/−^ (*n* = 57 cells, N = 3 mice) and MCU^+/+^ (*n* = 52 cells, N = 3 mice). Here and in the right panel data are presented as the mean value ± standard error of the mean. Dotted lines under the traces (composed of small asterisks) indicate the periods (from 60 to 290 s following CCK addition for NADH and from 21 to 420 s for FAD^+^) when the measurements from MCU^+/+^ and MCU^−/−^ cells were significantly different (*p* < 0.05). The right panel shows the ratio of NAD(P)H to FAD^+^ (calculated for the data shown in the left panels). Dotted line under the traces (composed of small asterisks) indicates the period (from 24 to 420 s following CCK addition) when the measurements from MCU^+/+^ and MCU^−/−^ cells were significantly different (*p* < 0.05). Figure S3 illustrates the differences in the maximal changes of NAD(P)H, FAD^+^ and the ratio of NAD(P)H to FAD^+^. (**B**) Measurements of oxygen consumption. The results of measurements are expressed as mean values ± standard errors (N = 6 mice for both MCU^+/+^ and MCU^−/−^). Changes in oxygen consumption rates (OCR) in CCK-stimulated pancreatic acinar cells from MCU^+/+^ mice are shown by blue trace (left) and from MCU^−/−^ mice by red trace (middle). OCR measurements in unstimulated cells are shown by cyan trace for MCU^+/+^ (left) and pink trace for MCU^−/−^ (middle). Paired t-test was used for comparing the OCR in CCK-stimulated and unstimulated cells (asterisks indicate *p* < 0.05). Note the smaller OCR response to CCK in MCU^−/−^ cells (see Figure S4 for further analysis of the CCK responses). Expanded fragments of OCR responses to CCK of acinar cells from MCU^+/+^ (blue) and MCU^−/−^ mice (red) are shown in the right panel. An independent t-test was used to compare the OCR responses in CCK-stimulated and unstimulated cells (asterisks indicate *p* < 0.05). CCK-stimulated and unstimulated cells were also subjected to the mitochondrial respiratory function test (Mito Stress Test) using sequential applications of oligomycin (O), carbonyl cyanide-4-trifluoromethoxy phenylhydrazone (FCCP), antimycin (A) and rotenone (R). There was no statistically significant difference between unstimulated MCU^+/+^ and MCU^−/−^ cells in the basal respiration (*p* = 0.68) or parameters determined by the Mito Stress Test: OCR linked to ATP production (*p* = 0.57), proton leak (*p* = 0.08), maximal respiration (*p* = 0.57) and spare respiratory capacity (*p* = 0.58). CCK stimulation did not produce resolvable changes of these parameters in both MCU^−/−^ and MCU^+/+^ cells (presumably because CCK responses were largely completed before the beginning of the Mito Stress Test). (**C**) Apoptosis in pancreatic acinar cells (left panel) isolated from MCU^−/−^ and MCU^+/+^ mice was assessed as the fluorescence of Caspase 3/7. The bar graph shows the fluorescence measurements divided by the fluorescence recorded in vehicle control experiment from the cells isolated from MCU^+/+^ mice. The bars indicate the mean values of measurements ± the standard errors. In this part of the study, five MCU^−/−^ mice and five MCU^+/+^ were used for experiments involving 200 µM TLCS and the same numbers utilized for the corresponding control experiments; five MCU^−/−^ mice and five MCU^+/+^ mice were used for experiments involving 1 nM CCK and the same numbers of mice utilized for the corresponding control experiments; and nine MCU^−/−^ mice and nine MCU^+/+^ mice were used for experiments involving 100 µM POA and the same numbers of mice were utilized in the corresponding control experiments. Necrosis in pancreatic acinar cells (right) isolated from MCU^−/−^ and MCU^+/+^ mice was assessed as the fluorescence of propidium iodide for experiments with CCK and POA (and their corresponding vehicle controls) and the fluorescence of SytoxRed for TLCS (and its corresponding vehicle control). The bar graph shows the fluorescence measurements divided by the fluorescence recorded in vehicle control experiments from the cells isolated from MCU^+/+^ mice. The bars indicate the mean values of measurements ± the standard errors. In this part of the study, five MCU^−/−^ mice and five MCU^+/+^ nice were used for experiments involving 200 µM TLCS and the same numbers utilized for the corresponding control experiments; five MCU^−/−^ mice and five MCU^+/+^ mice were used for experiments involving 1 nM CCK and the same numbers of mice utilized for the corresponding control experiments; and ten MCU^−/−^ mice and ten MCU^+/+^ mice were used for experiments involving 100 µM POA and the same numbers of mice utilized in the corresponding control experiments.

Mitochondrial Ca^2+^ is an important regulator of the TCA cycle (reviewed in [42,65,66]). Changes in NADH and FAD+ fluorescence can be utilized as indicators of the TCA activity in mitochondria of intact cells (e.g., [11,12,55]). The acinar cells derived from MCU^−/−^ animals displayed much smaller NADH and FAD^+^ responses to CCK than the cells from WT mice (Figure 2A and Figure S3). Our observation that mitochondrial Ca^2+^ responses to CCK stimulation of the acinar cells was drastically reduced in MCU^−/−^ cells is consistent with the results of NADH/FAD^+^ measurements (see Figure 2A and Figure S3). This finding was also consistent with the observed reduction in the CCK-stimulated oxygen consumption rate (Figure 2B and Figure S4). 

We next quantified pancreatic acinar cell death in vitro under pathological stimulation. All AP inducers caused increases in necrosis and apoptosis in the acinar cells derived from WT and MCU^−/−^ mice (Figure 2C). Notably, all mean values of parameters characterizing apoptosis and necrosis were reduced in MCU^−/−^ cells (in comparison with WT counterparts) for all tested AP inducers; however, the observed reductions were modest and the difference was statistically significant only for TLCS-induced necrosis (Figure 2C). The moderate protection against TLCS-induced necrosis and the reduction in mean values of measurements characterizing apoptosis and necrosis induced by CCK and POA (albeit not reaching statistical significance) suggests that MCU KO could offer some limited protection against the damaging cellular effects of AP inducers. This protection is, however, clearly incomplete, suggesting the redundancy of the injurious processes triggered by the AP inducers. 

### 3.2. Knockout of the Mitochondrial Calcium Uniporter Does not Reduce Severity of Experimental Acute Pancreatitis

**Figure 3:**
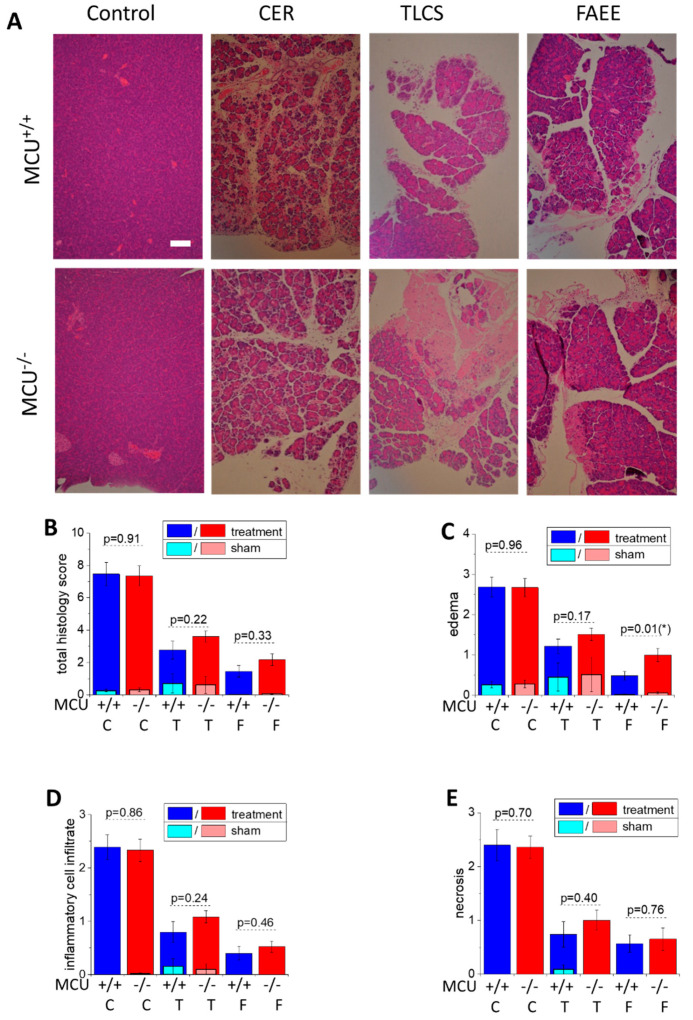
*MCU* knockout does not reduce histopathology scores in CER-AP, TLCS-AP and FAEE-AP. (**A**) Representative images of pancreatic sections demonstrating histopathology changes (hematoxylin and eosin staining; scale bar represents 100 µm). The models of AP are abbreviated on the graph as C for CER-AP, T for TLCS-AP and F for FAEE-AP. One representative control experiment (for CER-AP) is shown in this part of the figure; all saline controls had low total histopathology scores (see below). (**B**–**E**) The histopathology scores. The bars indicate the mean values ± the standard errors. In all parts of this figure, dark bars represent the treatment groups, while the light bars represent control (sham) groups (see Materials and Methods Section and text boxes above the bars specifying the color coding). There were no statistically significant differences between the control/sham groups of MCU^−/−^ and MCU^+/+^ mice in the histopathology scores for any type of control/sham experiments (*p* > 0.05). The scores for all treatment groups were significantly different (*p* < 0.05) from the scores for the corresponding control (sham) groups. (**B**) The total histopathology score for MCU^−/−^ and MCU^+/+^ mice. (**C**–**E**) The component scores: (**C**) edema (asterisk indicates p < 0.05); (**D**) inflammatory cell infiltration; and (**E**) necrosis. The p values for comparison between MCU^−/−^ and MCU^+/+^ mice in AP models are indicated above the corresponding bars. In this part of the study, 12 MCU^−/−^ and 13 MCU^+/+^ mice were used for the CER-AP; 7 MCU^−/−^ and 10 MCU^+/+^ for the TLCS-AP; and 8 MCU^−/−^ and 6 MCU^+/+^ for the FAEE-AP. Histopathological scores for AP induced in MCU^−/−^ and MCU^+/+^ did not reveal significant differences except for edema in FAEE-AP, which had a higher score for MCU^−/−^ (*p* = 0.01; marked with asterisk on the figure).

In this part of the study, we investigated the effect of genetic MCU ablation in three animal models of acute pancreatitis: caerulein hyperstimulation (CER-AP), retrograde perfusion of the pancreatic duct with bile acid TLCS (TLCS-AP), and fatty acid plus ethanol stimulation (FAEE-AP). Characteristic changes of parameters signifying the induction of AP in MCU^−/−^ and WT (MCU^+/+^) mice were observed in all three models of the disease (Figure 3). Pancreata of animals from all treatment groups displayed statistically significant increases (in comparison with corresponding sham treatment groups) of the overall histopathological score (Figure 3B) and its subclass scores: edema (Figure 3C), inflammatory cell infiltration (Figure 3D) and acinar cell necrosis (Figure 3E). However, in all three in vivo models, there were no significant differences between MCU^−/−^ and WT mice in the majority of histopathological parameters defining the severity of acute pancreatitis (Figure 3) The exception was the score for edema (Figure 3C), which was larger in MCU^−/−^ mice in conditions of FAEE-AP. 

**Figure 4:**
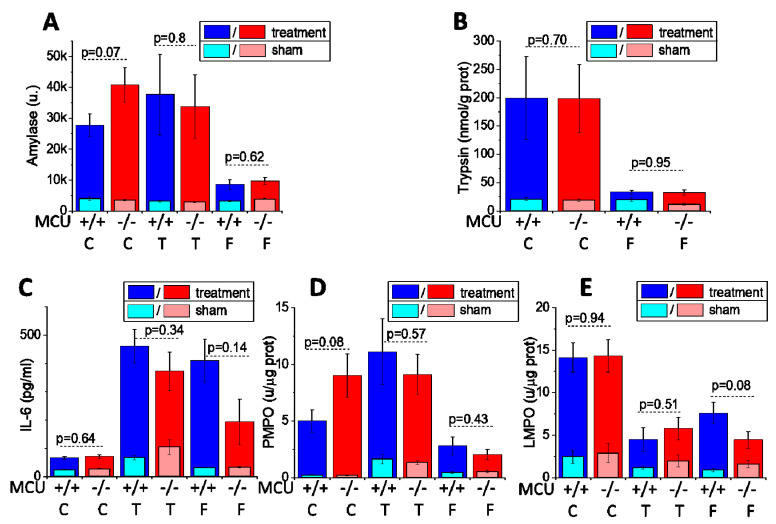
*MCU* knockout does not reduce biochemical parameters characterizing the severity of acute pancreatitis in CER-AP, TLCS-AP and FAEE-AP. The models of AP are abbreviated on the graph as C for CER-AP, T for TLCS-AP and F for FAEE-AP. The bars indicate the mean values ± the standard errors. In all parts of this figure dark, bars represent the treatment groups, while the light bars represent control (sham) groups (see Materials and Methods Section and text boxes above the bars specifying the color coding). There were no statistically significant differences between the control/sham groups of MCU^−/−^ and MCU^+/+^ mice in the values of biochemical parameters for any type of control/sham experiments (*p* > 0.05). The measured values of the biochemical parameters for all treatment groups were significantly different (*p* < 0.05) from the values for the corresponding control (sham) groups. The biochemical markers reflecting the severity of experimental AP are: (**A**) serum amylase; (**B**) pancreatic trypsin activity; (**C**) serum IL-6; (**D**) pancreatic myeloperoxidase activity; and (**E**) lung myeloperoxidase activity. The p values for comparison between MCU^−/−^ and MCU^+/+^ mice in AP models are indicated above the corresponding bars. In this part of the study, 12 MCU^−/−^ and 13 MCU^+/+^ mice were used for the CER-AP (with exception of pancreatic trypsin measurements for CER-AP, which were recorded on 7 MCU^−/−^ and 7 MCU^+/+^); 7 MCU^−/−^ and 10 MCU^+/+^ for the TLCS AP; and 8 MCU^−/−^ and 6 MCU^+/+^ for the FAEE-AP.

Consistently with the results of histopathological measurements, serum amylase (Figure 4A), pancreatic trypsin activity (Figure 4B), serum IL-6 (Figure 4C), pancreatic myeloperoxidase activity (Figure 4D) and lung myeloperoxidase activity (Figure 4E) were not significantly different (*p* > 0.05) in the experimental AP models involving MCU^−/−^ and WT mice (Figure 4A–E). 

The results of these experiments demonstrate that MCU knockout had no resolvable effect on the histopathological and biochemical parameters characterizing the severity of acute pancreatitis in the three experimental animal models of this disease.

## 4. Discussion

The primary aim of this study was to characterize the effect of MCU KO on the severity of experimental acute pancreatitis. The efficiency of constitutive MCU ablation was determined by Western blot analysis and confirmed by functional measurements of the changes in mitochondrial Ca^2+^ levels. Indeed, our experiments demonstrated that in pancreatic acinar cells Ca^2+^ entry in mitochondria upon various stimulations was drastically reduced in MCU^−/−^ animals. The prominent suppression of the [Ca^2+^]m response was observed for both TLCS and CCK. Interestingly, while the [Ca^2+^]m response to TLCS was completely abolished, CCK was still able to produce a drastically diminished but resolvable [Ca^2+^]m response in the cells from MCU^−/−^ mice. Importantly for this study, the observed strong inhibition of mitochondrial Ca^2+^ entry for both CCK and TLCS makes it highly unlikely that either agonist can trigger mitochondrial Ca^2+^ overload and mitochondrial Ca^2+^ toxicity in MCU^−/−^ cells. A tiny [Ca^2+^]m response to CCK in the cells from MCU^−/−^ mice could be explained by the reported non-MCU mitochondrial Ca^2+^ influx mechanisms [67,68,69]. The observed [Ca^2+^]m response was very small and difficult to investigate further. Importantly, our experiments demonstrated that MCU knockout drastically reduced mitochondrial Ca^2+^ entry. As expected, considering the wealth of literature on the subject (reviewed in [36,65,66]), the suppression of Ca^2+^ entry had a prominent inhibitory effect on stimulus-metabolism coupling (manifested by strong reduction of NADH/FAD^+^ responses to CCK stimulation). Our results are therefore consistent with the well-defined role of MCU in mediating Ca^2+^ entry [40,41] (reviewed in [70,71]) and the role of Ca^2+^-dependent dehydrogenases in the regulation of mitochondrial bioenergetics [36,65,66]. These results were consistent with substantial reduction in CCK-induced changes of oxygen consumption observed in the acinar cells from MCU^−/−^ mice. Notably, we have not observed resolvable differences in basal respiration or parameters determined by the Mito Stress Test (including maximal respiration and spare respiratory capacity) between the acinar cells isolated from MCU^−/−^ and MCU^+/+^ mice. These results are somewhat surprising but similar to the outcome of experiments on mouse embryonic fibroblasts from MCU^−/−^ and MCU^+/+^ mice [53]. MCU knockout had only a modest inhibitory effect on TLCS-induced acinar cell necrosis and did not cause significant inhibition of apoptosis or necrosis in cells treated with other AP inducers. The prominent role of mitochondrial Ca^2+^ overload in the initiation of acinar cell death was demonstrated in a number of studies [6,37,38,72]. Our finding was therefore unexpected, although consistent with the observation by Pan and colleagues from Finkel’s group that cell death of mouse embryonic fibroblasts (induced by a number of injurious stimuli including H_2_O_2_ and an inhibitor of ER Ca^2+^ pumps thapsigargin) is similar in the cells with and without MCU [53]. Our experiments revealed similar peak and plateau cytosolic Ca^2+^ responses to a supramaximal concentration of CCK in pancreatic acinar cells from MCU^−/−^ and MCU^+/+^ mice, suggesting that injurious effects associated with the cytosolic (i.e., non-mitochondrial) Ca^2+^ rise would also be similar in the cells from both types of animals. Such cytosolic components of Ca^2+^-induced damage could be the reason for the relatively modest differences in apoptosis and necrosis between the acinar cells from MCU^−/−^ and MCU^+/+^ mice found in our experiments. Activation of calpains [21], trypsinogen [14,73] and calcineurin [26] are amongst putative downstream processes which could potentially mediate injurious effects of cytosolic Ca^2+^. Further studies utilizing acinar cells from MCU^−/−^ mice could help to identify specific non-mitochondrial mechanisms of cytosolic Ca^2+^ toxicity.

Mitochondrial Ca^2+^ overload has been shown to trigger a non-specific increase in the permeability of the inner mitochondrial membrane (termed mitochondrial permeability transition (MPT)) mediated by the opening of mitochondrial permeability transition pore (MPTP) [74,75,76] (recently reviewed in [77,78]). Genetic inhibition of MPTP by global knock-out of cyclophilin D, which is a regulator of MPTP complex [79,80], was shown to protect animals against AP in all three models used in the present study [6]. Similar results were obtained with pharmacological cyclophilin D/MPTP inhibitors [81]. Mitochondrial Ca^2+^ entry is a prominent inducer of MPT in the acinar cells [37,38]. We therefore hypothesized that ablation of the MCU (primary mediator of mitochondrial Ca^2+^ entry) would reduce the severity of AP. Our finding that MCU knock-out does not protect against AP in vivo was unexpected but highlights the importance of mitochondrial function in reducing the severity of AP, as achieved by MPTP inhibition [6,81]. It should be noted that our results are similar to the observed lack of protection of cardiac tissue against ischemia-reperfusion in MCU^−/−^ mice [53] and MCU dominant negative mice [82]. It is conceivable that cell death mechanisms in MCU^−/−^ animals undergo adaptation, which reduces the relevance of mitochondrial Ca^2+^ overload and mitochondrial Ca^2+^ entry. One possible mechanism of such an adaptation is the recently reported decrease of MPTP sensitivity to [Ca^2+^]m in mitochondria isolated from MCU^−/−^ cells, permitting MPTP opening without a need for [Ca^2+^]m increase [83]. Another putative explanation could be provided by the multiplicity of the damaging mechanisms. Mitochondrial Ca^2+^ overload is not the only reported injurious effect triggered by the inducers of AP: activation of zymogens and aberrant release of digestive proteases (e.g., [28,29,30,84]), activation of calpains [21], disruption of trafficking and vacuolization [22,23,24,25,85] and aberrant autophagy [25,31,85,86] are just a few processes that could be responsible for acinar cell damage and AP initiation. The absence of a resolvable effect of MCU knockout on acinar cell death and the severity of AP might therefore be explained by the redundancy of injurious effects triggered by the AP inducers. On the other hand, our results reveal a clear and substantial contribution of MCU to the stimulus-metabolism coupling in pancreatic acinar cells and further studies on the role of the MCU in the regulation of digestion are warranted.

## Supplementary Materials

The following are available online at https://www.mdpi.com/2073-4409/9/6/1407/s1. Figure S1: Western blot analysis of pancreata from MCU^−/−^ and MCU^+/+^ mice and the distribution of MtRCaMP fluorescence in pancreatic acinar cells. Figure S2: Mitochondrial Ca^2+^ responses to palmitoleic acid (POA) in acinar cells isolated from MCU^−/−^ and MCU^+/+^ mice. Figure S3: Amplitudes of CCK-induced changes in NAD(P)H fluorescence, FAD fluorescence and NAD(P)H/FAD ratio in acinar cells isolated from MCU^−/−^ and MCU^+/+^ mice. Figure S4: Amplitudes of CCK-induced changes in oxygen consumption rates (OCR) and areas under the peaks of CCK responses.
