# Supplementary material for: Knockout of the Mitochondrial Calcium Uniporter Strongly Suppresses Stimulus-Metabolism Coupling in Pancreatic Acinar Cells but Does not Reduce Severity of Experimental Acute Pancreatitis
Source: Cells. 2020 Jun 5;9(6):1407. doi: 10.3390/cells9061407 (PMC7349284; doi:10.3390/cells9061407)
Supplement: Supplementary file 1 [file cells-09-01407-s001.pdf]

Supplemental information

Knockout of the mitochondrial calcium uniporter strongly suppresses stimulus-metabolism coupling in pancreatic acinar cells but does not reduce severity of experimental acute pancreatitis

Michael Chvanov[1], Svetlana Voronina[1], Xiaoying Zhang[2], Svetlana Telnova[1], Robert Chard[1], Yulin Ouyang[1], Jane Armstrong[2], Helen Tanton[1], Muhammad Awais[2], Diane Latawiec[2], Robert Sutton[2], David N. Criddle[1] and Alexei V. Tepikin[1]

[1]*Department of Cellular and Molecular Physiology, University of Liverpool, Liverpool, UK.*
[2]*Liverpool Pancreatitis Research Group, Royal Liverpool University Hospital, Institute of Translational Medicine, University of Liverpool, Liverpool, UK.*

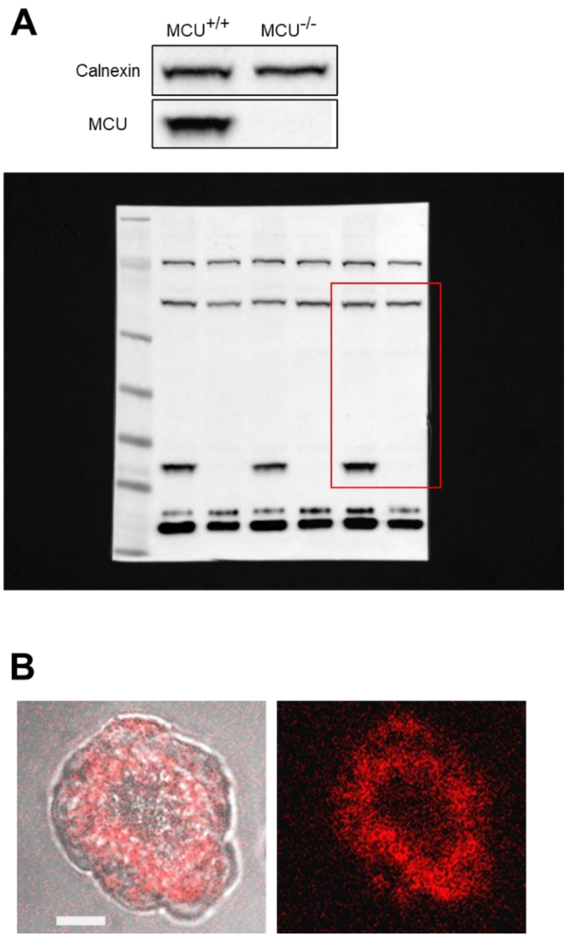

Figure S1. Western blot analysis of pancreata from *MCU*$^{-/-}$ and *MCU*$^{+/+}$ mice and the distribution of MtRCaMP fluorescence in pancreatic acinar cells. (**A**) Western blot analysis of pancreata from *MCU*$^{-/-}$ and *MCU*$^{+/+}$ mice. The figure shows the complete Western Blot that supplements Figure 1A and confirms the lack of MCU protein in a representative *MCU*$^{-/-}$ mouse. The fragments shown in the main manuscript were derived from the section

highlighted by the red box. (**B**) Images of MtRCaMP in a small cluster of *MCU*$^{-/-}$ pancreatic acinar cells showing a typical mitochondrial distribution. Right panel shows the distribution of fluorescence. Left panel shows the overlay of transmitted light and fluorescence. Scale bar represents 10 µm. A similar distribution was observed in the acinar cells from *MCU*$^{+/+}$ mice (see Figure 1B in the main part of the paper). Note clustering of mitochondria around the central granular region of the cluster (in both Figure 1B and this figure).

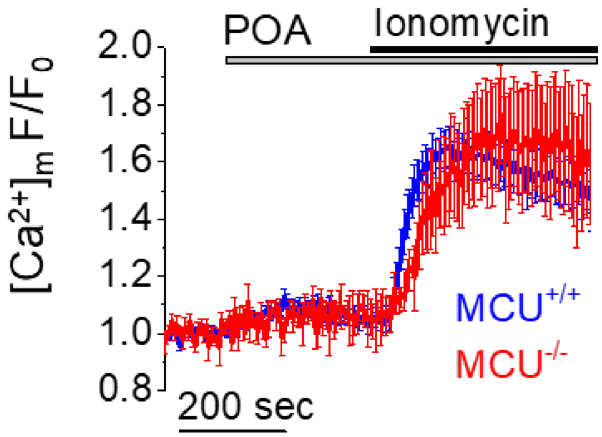

Figure S2. Mitochondrial Ca^{2+} responses to palmitoleic acid (POA) in acinar cells isolated from *MCU*$^{-/-}$ and *MCU*$^{+/+}$ mice. The traces show the fluorescence of MtRCaMP (F) normalized to its initial fluorescence (F_0) in acinar cells isolated from *MCU*$^{-/-}$ mice (n=5 cells, N=3 mice) and *MCU*$^{+/+}$ mice (n=30 cells, N= 3 mice). Each data point on the graph is presented as the mean value ± standard error of the mean. Responses to 100 µM POA were minor; there was no resolvable difference. The application of 100 µM POA was followed by 20 µM Ionomycin/10 mM $CaCl_2$.

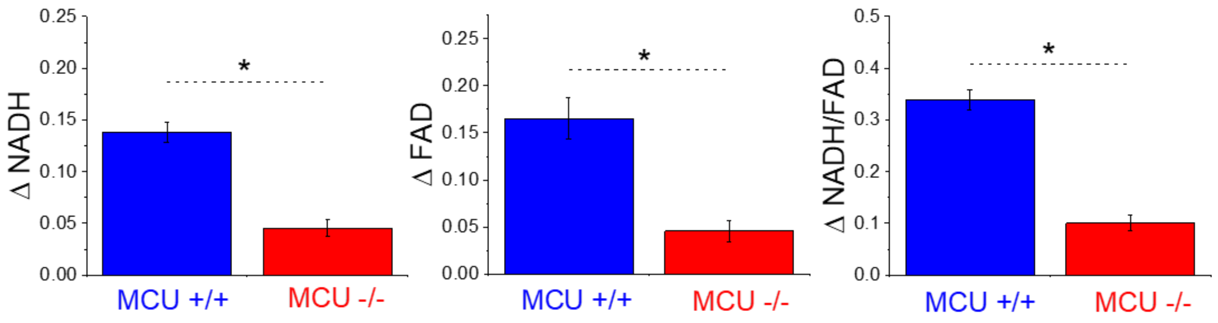

Figure S3. Amplitudes of CCK-induced changes of NAD(P)H fluorescence, FAD fluorescence and NAD(P)H / FAD ratio in acinar cells isolated from *MCU*$^{-/-}$ and *MCU*$^{+/+}$ mice. The figure

supplements Figure 2A. The amplitudes (Δ NAD(P)H, Δ FAD and Δ NAD(P)H / FAD) were determined as the maximal differences between the values of these parameters attained after CCK application (during the recording period shown in Figure 2A) and the values just before CCK application. The graph shows absolute values of the amplitudes. All CCK-induced responses were significantly different ($p < 0.05$, indicated by asterisks on the graph) in the cells from $MCU^{+/+}$ and $MCU^{-/-}$ mice.

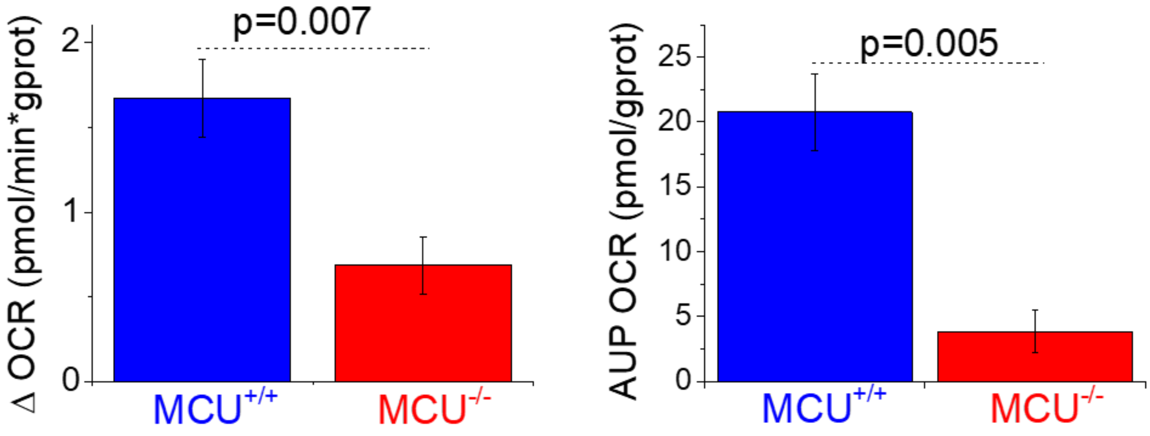

Figure S4. Amplitudes of CCK-induced changes in oxygen consumption rates (OCR) and areas under the peaks of CCK responses. This figure supplements Figure 2B of the main manuscript and compares CCK responses in cells isolated from $MCU^{+/+}$ and $MCU^{-/-}$ mice. The amplitude of OCR response to CCK stimulation was determined as the difference (Δ OCR) between the maximal OCR of CCK-stimulated cells (first OCR measurement after the CCK application, see Figure 2B) and the corresponding OCR (i.e. measured at the same time point) of unstimulated cells. An independent-samples t-test was used to compare the amplitudes of responses to CCK (N=6 mice). An independent t-test was also used to compare areas under the peaks (AUP OCR) of CCK responses (determined as areas between the OCR curves obtained from CCK-stimulated and unstimulated cells and limited by the time points at which the OCR of CCK-stimulated cells was statistically larger than the OCR of unstimulated cells). The difference between $MCU^{-/-}$ and $MCU^{+/+}$ cells was statistically significant for both Δ OCR and AUP OCR (the p values are shown above the bars of the bar graphs).
